# [^18^F]FET-PET in children and adolescents with central nervous system tumors: does it support difficult clinical decision-making?

**DOI:** 10.1007/s00259-023-06114-6

**Published:** 2023-01-21

**Authors:** Olivia Kertels, Jürgen Krauß, Camelia Maria Monoranu, Samuel Samnick, Alexander Dierks, Malte Kircher, Milena I. Mihovilovic, Mirko Pham, Andreas K. Buck, Matthias Eyrich, Paul-Gerhardt Schlegel, Michael C. Frühwald, Brigitte Bison, Constantin Lapa

**Affiliations:** 1 Institute of Diagnostic and Interventional Radiology, University Hospital Würzburg, Oberdürrbacher Strasse 6, 97080 Würzburg, Germany; 2 Section Pediatric Neurosurgery, Department of Neurosurgery, University Hospital Würzburg, Oberdürrbacher Strasse 6, 97080 Würzburg, Germany; 3 Department of Neuropathology, Institute for Pathology, University of Würzburg, Josef-Schneider-Strasse 2, 97080 Würzburg, Germany; 4 Department of Nuclear Medicine, University Hospital Würzburg, Oberdürrbacher Strasse 6, 97080 Würzburg, Germany; 5 Nuclear Medicine, Faculty of Medicine, University of Augsburg, Stenglinstrasse 2, 86156 Augsburg, Germany; 6 Institute of Diagnostic and Interventional Neuroradiology, University Hospital Würzburg, Josef-Schneider-Str. 11, 97080 Würzburg, Germany; 7 Department of Pediatric Hematology, Oncology and Stem Cell Transplantation, University Children’s Hospital, University of Würzburg, Josef-Schneider- Str. 2, 97080 Würzburg, Germany; 8 Paediatric and Adolescent Medicine, University Medical Center Augsburg, Stenglinstrasse 2, 86156 Augsburg, Germany; 9 Diagnostic and Interventional Neuroradiology, Neuroradiological Reference Center for Pediatric Brain Tumor (HIT) Studies of the German Society of Pediatric Oncology and Hematology, Faculty of Medicine, University of Augsburg, Stenglinstr. 2, 86156 Augsburg, Germany

**Keywords:** [^18^F]FET, PET, Positron emission tomography, Pediatric brain tumor

## Abstract

**Purpose:**

Positron emission tomography (PET) with O-(2-[^18^F]fluoroethyl)-L-tyrosine ([^18^F]FET) is a well-established tool for non-invasive assessment of adult central nervous system (CNS) tumors. However, data on its diagnostic utility and impact on clinical management in children and adolescents are limited.

**Methods:**

Twenty-one children and young adults (13 males; mean age, 8.6 ± 5.2 years; range, 1–19 at initial diagnosis) with either newly diagnosed (*n* = 5) or pretreated (*n* = 16) CNS tumors were retrospectively analyzed. All patients had previously undergone neuro-oncological work-up including cranial magnetic resonance imaging. In all cases, [^18^F]FET-PET was indicated in a multidisciplinary team conference. The impact of PET imaging on clinical decision-making was assessed. Histopathology (*n* = 12) and/or clinical and imaging follow-up (*n* = 9) served as the standard of reference.

**Results:**

The addition of [^18^F]FET-PET to the available information had an impact on further patient management in 14 out of 21 subjects, with avoidance of invasive surgery or biopsy in four patients, biopsy guidance in four patients, change of further treatment in another five patients, and confirmation of diagnosis in one patient.

**Conclusion:**

[^18^F]FET-PET may provide important additional information for treatment guidance in pediatric and adolescent patients with CNS tumors.

**Supplementary Information:**

The online version contains supplementary material available at 10.1007/s00259-023-06114-6.

## Introduction

Primary tumors of the central nervous system (CNS) are the most common solid malignancies in childhood and one of the main causes for cancer-related deaths in children, with 5-year overall survival rates of up to 75% depending on risk group and histology [1–3]. Since childhood brain tumors comprise various biological entities (astrocytoma, medulloblastoma, and ependymoma being the most common) with rather heterogeneous presentation (depending on the tumor location and the age of the child), early diagnosis remains challenging [4]. Cranial magnetic resonance imaging (cMRI) is considered the standard of care for neuroimaging. It provides detailed anatomical and structural information as well as characteristic features of different tumor entities. Although advanced cMRI techniques (such as diffusion tensor imaging, perfusion imaging, or spectroscopy) are able to deliver additional information, it remains a diagnostic challenge to specify tumor grading and differentiate between true progression and therapy-related findings [5].

Positron emission tomography (PET) using O-(2-[^18^F]fluoroethyl)-L-tyrosine ([^18^F]FET) as a marker of amino acid transport can offer supplemental information regarding tumor biology. It is an established tool in adult brain tumor imaging, where it has proven valuable in prognostication [6–8], treatment monitoring [9–11], and differentiation of non-specific post-therapeutic changes (pseudoprogression) from tumor recurrence [12–14]. To date, only very few studies have focused on the use and utility of [^18^F]FET-PET in pediatric brain tumors. This study aimed at investigating the additional benefit of [^18^F]FET-PET in children and adolescents with primary CNS tumors in a real-world scenario. To this end, we retrospectively analyzed the clinical impact of amino acid PET in cases requiring a complex clinical process of decision-making (i.e., due to insufficient or equivocal information gained from cMRI).

## Material and methods

### Subjects

This retrospective analysis included 21 consecutive pediatric and adolescent patients with primary CNS tumors (mean age, 8.6 ± 5.2 years; age range, 1–19 at initial diagnosis; and mean age, 12.6 ± 5.6; age range, 2–23 at PET scan date) who underwent [^18^F]FET-PET for further diagnostic work-up between July 2010 and October 2018 at the Department of Nuclear Medicine at University Hospital Würzburg, Germany. The general cut-off for patient age was 18 at initial diagnosis. One older patient was included as she was diagnosed with a typical pediatric brain tumor (pilomyxoid astrocytoma of the optic pathway) at the age of 19 (patient #11). Five patients presented with newly diagnosed gliomas, while the remaining 16 subjects with primary brain tumors were referred due to equivocal MRI diagnosis.

All subjects had previously undergone comprehensive neuro-oncologic work-up as described in more detail in sections “cMRI” and “Standard of reference.” In these select cases, [^18^F]FET-PET was also clinically indicated at a weekly multidisciplinary team conference in pediatric neuro-oncology for the confirmation of diagnosis or determination of further treatment planning (e.g., extent of irradiation or biopsy guidance). This was conducted due to difficulties in the clinical decision-making based on the available information (all clinical and histopathological data in addition to current MR imaging) alone, and the expert panel opted for additional PET imaging in order to gain clinical confidence.

Written informed consent was provided by all legal guardians or patients, respectively. The study adhered to the standards established in the Declaration of Helsinki. Given the retrospective nature of this analysis of routinely acquired data, the local ethics committee of the University of Würzburg waived the need for further approval.

### Preparation of [^18^F]FET and PET imaging

[^18^F]FET was synthetized in-house at the Interdisciplinary PET Centre of the University Hospital of Würzburg using a GE TRACERlab FX-FN synthesis module (GE Medical Systems, Uppsala, Sweden) as previously described [13, 15]*.*

All patients fasted for at least 12 h prior to PET imaging. Twenty minutes after intravenous injection of [^18^F]FET (156 ± 69 MBq), patients were scanned on an integrated PET/CT scanner (Biograph mCT 64, Siemens Healthineers, Knoxville, TN). Static PET emission data were collected in three-dimensional mode using a 200 × 200 matrix for 10 min. Subsequent CT scans for attenuation correction were acquired using a low-dose protocol (CARE Dose 4D; 80 mAs; 120 kV; matrix, 512 × 512; 3 mm slice thickness; increment, 30 mm/s; rotation time, 0.5 s; pitch index, 0.8). PET images were reconstructed iteratively (TrueX; 3 iterations; 24 subsets; Gaussian filtering, 2 mm; decay, attenuation and scatter correction) using dedicated manufacturer software (syngo MI.PET/CT; Siemens Healthineers).

### cMRI

All patients underwent a cranial MRI prior to [^18^F]FET-PET, with a median interval of 18 days between PET and cMRI (range, 5–70 days). cMRI was performed in-house for 18 out of 21 patients. A total of 10/18 patients were scanned on a 1.5 T MRI (Magnetom Symphony or Magnetom Aera, both Siemens Healthcare, Erlangen, Germany) and 8/18 patients on a 3 T MRI (Magnetom Trio Tim, Siemens Healthcare, Erlangen, Germany). The remaining 3 patients presented with cMRI studies from other hospitals.

Basic cMRI, including T2-weighted images (T2WI), fluid-attenuated inversion recovery (FLAIR) (in 18/21 patients; proton density images in the remaining cases), T1-weighted images without (T1WI) as well as with contrast enhancement (T1WI + CE), and diffusion weighted images (DWI) were obtained for all patients but one (patient #20, missing diffusion weighted imaging). Additional spectroscopy was available in 5/21 subjects.

High-grade gliomas with high cellularity were defined by a low signal on T2WI and restricted diffusion. In contrast, low-grade gliomas displayed signs of low cellularity with a high signal on T2WI and high apparent diffusion coefficient values. Details of the cMRI examinations are summarized in Supplemental Table 1.

### PET image analysis

All [^18^F]FET-PET scans were evaluated independently by one very experienced investigator (rater 1, AKB, more than 15 years of experience) and one with intermediate experience (rater 2, OK, more than 5 years of experience). Distinction between tumor and non-specific tracer uptake was based on combined analysis of [^18^F]FET-PET and MR, previous imaging, and clinical history and as previously described [16].

First, a visual inspection of scans for tumor uptake was performed. Then, on the axial slice presenting the maximum tumor uptake, regions of interest (ROI) were selected. Standardized uptake values for maximal (SUV_max_) and mean tumor uptake (SUV_mean_) were derived by placing a 10-mm circular region of interest over the area with the peak activity. For assessment of background activity, normal-appearing brain on the contralateral hemisphere (SUV_BKG_) was selected, and data evaluation including calculation of tumor-to-background ratios (TBR) was performed as previously described [17–19]. Subsequently, mean and maximum tumor-to-background ratios (TBR_mean_; TBR_max_) were calculated. For the differentiation of vital tumor from unspecific changes, validated TBR cut-off values were used [13, 18].

The radiotracer concentration in the ROIs was normalized to the injected dose per kilogram of patient’s body weight to derive the SUVs.

### Assessment of the clinical impact of [^18^F]FET-PET

Individual patient cases, including cMRI and [^18^F]FET-PET scans, were subsequently discussed by a multidisciplinary team. The clinical impact of [^18^F]FET-PET on the treatment was rated in a multidisciplinary consensus by an experienced pediatric neurosurgeon (JK), experienced pediatric oncologist (PGS), and two nuclear medicine specialists (AKB, CL) who had access to all clinical data but were blinded to future treatment decisions and outcomes. Impact on clinical decision-making due to addition of [^18^F]FET-PET to the diagnostic algorithm was rated with a two-sided score (yes versus no), with impact being defined as a direct influence on patient management by the addition of relevant new, treatment-guiding information (e.g., detection of residual tumor, differentiation of true tumor progression versus treatment-related changes, change in definition of tumor extent, or change of biopsy location).

### Standard of reference

The reference standard was biopsy or resection, if feasible (*n* = 12). Otherwise, a combination of clinical and radiological follow-up was used (*n* = 9). Twelve patients underwent either serial stereotactic biopsy or surgery for histopathological analysis. Histological classification, molecular genetic analysis, and tumor grading were accomplished by an experienced neuropathologist (CMM). The biopsy samples/surgical specimens were fixed in formalin and embedded in paraffin. All samples (3–4 μm sections stained with hematoxylin and eosin) were histologically assessed and graded according to the respective current WHO criteria [20, 21].

Nine patients received serial follow-up cMRI that served as radiological standard of reference. Pre- and untreated low-grade tumors with stable tumor lesions during follow-up were rated as remnant tumor. For high-grade tumors, stable lesions for 6 months without treatment were classified as non-tumors.

### Statistical analysis

Most of the data provided are descriptive. Descriptive statistics for patient characteristics were reported as mean ± standard deviation (SD), median, and range.

## Results

### Patient characteristics

A total of 21 pediatric and adolescent patients with primary CNS tumors were included (mean age, 8.6 ± 5.2 years; median age 8; range, 1–19 at initial diagnosis; and mean age, 12.6 ± 5.6; median age 14; range, 2–23 at PET scan date). PET and MRI scan were performed within a median of 14 days (range, 5–70 days). Five patients presented with newly diagnosed gliomas, and sixteen children/adolescents were referred with pretreated brain tumors (3 patients with surgery alone, 7 patients with combined surgery and radiochemotherapy, 2 patients with combined surgery and chemotherapy, 3 patients with radiochemotherapy, and 1 patient with chemotherapy alone). In patients who had received combined radiochemotherapy, [^18^F]FET-PET scans were performed more than 12 weeks from cessation of radiotherapy (median, 31 months; range, 3–141 months).

Six patients presented with low-grade gliomas (pretreated WHO III anaplastic astrocytoma, at the time point of imaging graded as WHO I pilocytic astrocytoma, *n* = 1; WHO grade I pilocytic astrocytoma, *n* = 2; WHO grade II pilomyxoid astrocytoma/optic pathway glioma, *n* = 1; radiological diagnosis of low-grade glioma, *n* = 2) and 15 subjects with high-grade tumors, distributed as follows: WHO grade III anaplastic astrocytoma (*n* = 4, one of them as second malignancy after initial treatment of a germ cell tumor), WHO grade III anaplastic ependymoma (*n* = 1), WHO grade IV diffuse intrinsic pontine glioma (DIPG, *n* = 2) (neuroradiological diagnosis, one as a second malignancy after initial treatment of a medulloblastoma), WHO grade IV CNS primitive neuroectodermal tumor (*n* = 1), WHO grade IV ependymoblastoma (*n* = 2), and WHO grade IV glioblastoma (*n* = 5). Detailed patient characteristics are summarized in Table 1.

**Table 1:** Patient characteristics

	Sex	Age at ID/PET	Tumor entity (at time point of PET)	Localization (side)	WHO grade	Initial diagnosis (ID)/follow-up (FU)	Prior therapy
1	M	3/3	Ependymoblastoma	Frontal/temporal lobe (right)	IV	FU	Surgery + CTx
2	F	7/8	GBM	Thalamus/crus cerebri (left)	IV	FU	Surgery + RCTx
3	F	3/11	DIPG	Pons	IV	FU	RCTx
4	M	8/9	LGG	Frontal/parietal lobe (right)	n/a	ID	None
5	F	13/14	Anaplastic ependymoma	Thalamus (both)	III	FU	RCTx
6	M	15/15	LGG	Frontal lobe (left)	n/a	ID	None
7	M	7/7	GBM	Frontal/temporal lobe (right)	IV	ID	None
8	F	2/4	Ependymoblastoma	Frontal lobe (right)	IV	FU	Surgery + RCTx
9	M	14/14	Anaplastic astrocytoma/GC	Frontal (both), temporal lobe (right)	III	FU	Surgery + RCTx
10	M	13/23	Anaplastic astrocytoma/GC	Frontal (both)/temporal lobe, thalamus (left)	III	FU	RCTx
11	F	19/22	PMA/ OPG	Chiasm/hypothalamus	II	FU	Surgery
12	M	14/14	GBM	Thalamus/hypothalamus (left)	IV	FU	Surgery
13	F	8/17	Pilocytic astrocytoma/Tectumglioma	Tectum	I	FU	Surgery
14	M	8/15	DIPG (second malignancy, initially medulloblastoma)	Pons	IV	FU	Surgery + RCTx
15	M	11/11	GBM	Temporal lobe (right)	IV	FU	Surgery + RCTx
16	M	2/14	PNET	Frontal lobe (right)	IV	FU	Surgery + RCTx
17	M	8/10	Anaplastic astrocytoma	Thalamus (both)	III	ID	None
18	F	1/2	Pilocytic astrocytoma/OPG (initially anaplastic pilocytic astrocytoma)	Chiasm/hypothalamus	I (initially III)	FU	CTx
19	F	2/14	Pilocytic astrocytoma	Tectum/cerebellum	I	FU	Surgery + CTx
20	M	17/17	GBM/ GC	Frontal (both), temporal lobe (right)	IV	ID	None
21	M	6/20	Anaplastic astrocytoma (second malignancy, initially GCT)	Frontal lobe (left)	III	FU	Surgery + RCTx

*CTx*, chemotherapy; *DIPG*, diffuse intrinsic pontine glioma; *GBM*, glioblastoma multiforme; *GC*, gliomatosis cerebri; *GCT*, germ cell tumor; *HGG*, high-grade glioma; *ID*, initial diagnosis; *LGG*, low-grade glioma; *MRI*, magnetic resonance imaging; *n/a*, not available; *OPG*, optic pathway glioma; *PMA*, pilomyxoid astrocytoma; *PNET*, primitive neuroectodermal tumor; *RCTx*, radiochemotherapy

### PET findings

Fourteen out of 21 patients were PET-positive (3/5 with newly diagnosed brain tumors, 11/16 with pretreated lesions).

In the patients with newly diagnosed CNS tumors, SUV_mean_ and SUV_max_ ranged from 1.1 to 3.6 and from 1.3 to 5.0; TBR_mean_ and TBR_max_ ranged from 0.8 to 3.8 and from 1.0 to 5.1, respectively. All patients were correctly classified as high-grade (*n* = 3/3) or low-grade glioma (*n* = 2/2).

In the patients with concern of tumor recurrence or persistence of vital tumor, SUV_mean_ and SUV_max_ ranged from 1.5 to 5.4 and from 1.7 to 6.1, respectively. While TBR_mean_ and TBR_max_ ranged from 1.1 to 3.9 and from 1.2 to 4.7, respectively. The diagnostic accuracy of [^18^F]FET-PET in this sub-cohort was 88% (*n* = 14/16) with a sensitivity of 100% (*n* = 11/11) and a specificity of 60% (*n* = 3/5). Individual imaging results are presented in Supplemental Table 2.

### Impact of additional [^18^F]FET-PET imaging on clinical decisions

**Fig. 1:**
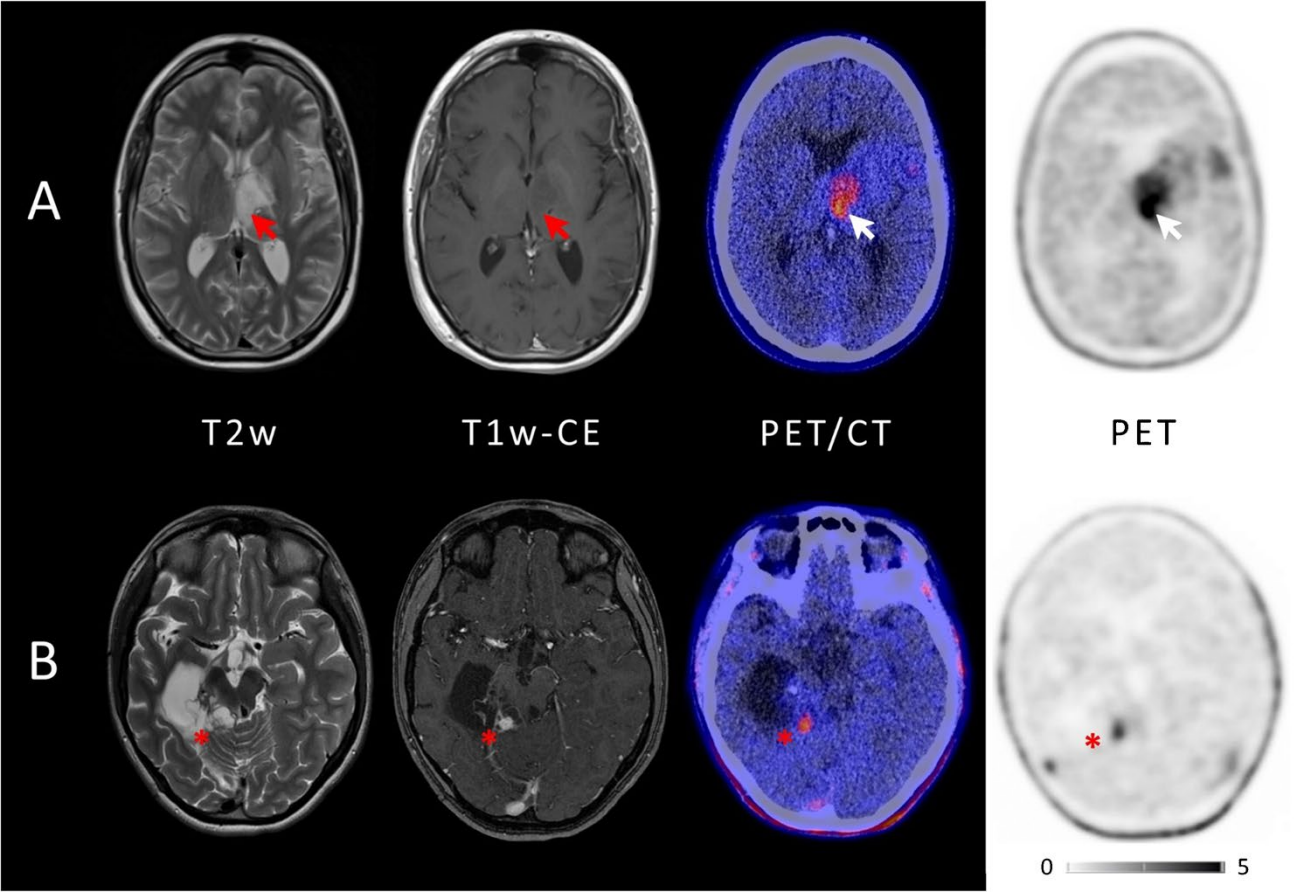
Example of two patients with concordant imaging results (**A**) and incremental diagnostic value of [^18^F]FET-PET (**B**). **A** Example of a patient (#10) with an anaplastic astrocytoma WHO grade III who was treated with combined radiochemotherapy prior to PET. Axial T2WI and T1WI + CE depict the tumor in the left thalamus and insular region (red arrows); axial PET shows high uptake of [^18^F] FET in these regions (white arrows). Both MRI and PET identified correctly the tumor recurrence. **B** Example of a patient (#19) with a pilocytic astrocytoma who was treated with surgery and chemotherapy prior to PET. Axial T2WI and T1WI + CE show cystic as well as contrast enhancing lesions next to the surgical cavity consistent with both unspecific changes and tumor recurrence (red asterisk); in contrast, axial PET demonstrates focal high uptake of [^18^F]FET (white asterisk)

[^18^F]FET-PET had an impact on further treatment decisions in a total of 14 out of 21 patients. Invasive surgery or biopsy was avoided in four patients, and PET was crucial to biopsy or surgery guidance in another four patients. In three patients, [^18^F]FET-PET directly changed further therapy: one patient received chemotherapy instead of radiotherapy (patient #2), chemotherapy was changed to another regimen in another patient (patient #5), and one patient received no further radiotherapy (patient #10). In one patient (patient #15), [^18^F]FET-PET confirmed the suspicion on true tumor progression and thus prompted subsequent surgery. A detailed overview of the individual clinical impact of [^18^F]FET-PET is presented in Table 2. Respective examples are given in Figure 1.

**Table 2:** Individual clinical impact of [^18^F]FET-PET

	Tumor entity at (time point of PET)	Initial diagnosis	Indication for PET	PET	Status after PET	Influence of PET on management	Follow-up	Confirmation of diagnosis
1	Ependymoblastoma	No	Residual tumor vs. infarct	Pos	Confirmation of vital tumor, suspicion for distant lesion	No surgery	(distant) PD	Clinical
2	GBM	No	SD vs. PD (new lesion)	Pos	Confirmation of PD	Chemotherapy instead of radiotherapy	PD (new lesion)	Clinical (new lesion)
3	DIPG	No	Post-therapeutic changes vs. vital tumor	Neg	Confirmation of post-therapeutic changes	None	Post-therapeutic changes	Clinical
4	LGG	Yes	HGG vs. LGG/biopsy guidance	Neg	No PET-positive tumor, results consistent with LGG	No biopsy	LGG	Clinical
5	Anaplastic ependymoma	No	PsPD vs. PD	Pos	Confirmation of PD	Change of chemotherapy	PD	Clinical
6	LGG	Yes	HGG vs. LGG/biopsy guidance	Neg	No PET-positive tumor, results consistent with LGG	No biopsy	LGG	Clinical
7	GBM	Yes	HGG vs. LGG/biopsy guidance	Pos	PET-positive tumor, biopsy guidance	Biopsy guidance	HGG	Histology
8	Ependymoblastoma	No	Post-therapeutic changes vs. PD	Neg	Confirmation of post-therapeutic changes	No surgery	Post-therapeutic changes	Clinical
9	Anaplastic astrocytoma/GC	No	Radiotherapy planning for suspected residual tumor	Pos	Confirmation of residual tumor	None	PD	Histology
10	Anaplastic astrocytoma/GC	No	Radiotherapy planning for suspected PD	Pos	Confirmation of multifocal, widespread PD	No further radiotherapy	PD	Clinical
11	PMA/ OPG	No	PsPD vs. PD	Pos	Confirmation of PD	None	PD	Histology
12	GBM	No	Radiotherapy planning for suspected PD	(False) neg	No PET-positive volume for radiotherapy planning	Watch and wait	PD	Histology
13	Pilocytic astrocytoma/Tectumglioma	No	Radiotherapy planning for suspected PD	Pos	Confirmation of PD, no change in scheduled radiotherapy	None	PD	Histology
14	DIPG (second malignancy, initially medulloblastoma)	No	Post-therapeutic changes vs. second malignancy	(False) neg	No PET-positive tumor, results consistent with post-therapeutic changes	Watch and wait	PD	Clinical
15	GBM	No	PsPD vs. PD	Pos	Confirmation of PD	Surgery	PD	Histology
16	PNET	No	Confirmation of diagnosis (cMRI consistent with cavernoma)	Neg	No PET-positive tumor, results consistent with cavernoma	None	Cavernoma	Histology
17	Anaplastic astrocytoma	Yes	Biopsy guidance	Pos	PET-positive tumor, biopsy guidance	Biopsy guidance	HGG	Histology
18	Pilocytic astrocytoma/OPG (initially anaplastic astrocytoma)	No	Biopsy guidance	Pos	PET-positive tumor, biopsy guidance	Biopsy guidance	PR (with therapy)	Histology
19	Pilocytic astrocytoma	No	PsPD vs. PD	Pos	Confirmation of vital tumor (no change in scheduled surgery)	None	PD	Histology
20	GBM/GC	Yes	Biopsy guidance	Pos	PET-positive tumor, results consistent with GBM (no biopsy, no change in scheduled surgery)	None	HGG	Histology
21	Anaplastic astrocytoma (second malignancy, initially GCT)	No	Post-therapeutic changes vs. second malignancy (if second malignancy: biopsy guidance)	Pos	PET-positive tumor, biopsy guidance	Biopsy guidance	PD	Histology

*DIPG*, diffuse intrinsic pontine glioma; *GBM*, glioblastoma; *GC*, gliomatosis cerebri; *GCT*, germ cell tumor; *HGG*, high-grade glioma; *LGG*, low-grade glioma; *Neg*, negative; *OPG*, optic pathway glioma; *PD*, progressive disease; *PMA*, pilomyxoid astrocytoma; *PNET*, primitive neuroectodermal tumor; *Pos*, positive; *PR*, partial response; *PsPD*, pseudoprogression; *SD*, stable disease

## Discussion

The value of [^18^F]FET-PET as an easy-to-read and robust tool in imaging for adults with gliomas has been established and has repeatedly been demonstrated over many years. Only a few studies have focused on its usefulness in and value for pediatric and adolescent patients with primary CNS tumors [22–26].

A recent prospective Danish study reported significantly increased diagnostic accuracy and clinical impact in 8% of scans when PET imaging was added to cMRI [25]. Notably, in cases deemed clinically indicated due to difficult decision-making on cMRI alone, its impact was as high as 33%.

Our study further supports these findings. Our real-world data suggest that [^18^F]FET-PET is a useful adjunct in challenging pediatric settings. In our cohort, inclusion of amino acid PET in the diagnostic algorithm impacted clinical management in two thirds of patients, either by avoiding surgery or biopsy, guiding targeted biopsy or surgery, changing further therapy management, or prompting alternative surgery. Consistent with the vast body of the literature available for adults, it reliably distinguished low-grade from high-grade glioma [27–29] and unspecific changes (e.g., (late) pseudoprogression, radiation necrosis) from true tumor recurrence [12, 14, 30].

cMRI remains the standard imaging modality in pediatric brain tumors, including medulloblastoma, atypical teratoid rhabdoid tumor, ependymoma, and primitive neuroectodermal tumor displaying characteristic imaging features. In times of molecular pathology, the diagnosis of diffuse intrinsic pontine glioma, optic pathway glioma, and tectal glioma is still based on neuroradiolological criteria. Additionally, in patients with DIPG and optic pathway glioma, it is still legitimate to begin therapy without previous biopsy if typical imaging criteria are fulfilled. T2W- and DW-imaging provides information about cellularity as a sign of aggressiveness in order to differentiate low-grade from high-grade gliomas. MR-based imaging in pediatric patients with central nervous system tumors is also useful for assessment of tumor dynamics (especially in low-grade gliomas, in order to initiate therapy), as well as treatment response monitoring and patient follow-up, due to the fact that residual tumor growth is exclusively detectable by sequential scans. However, despite the broad range of utility of this imaging modality, there remain challenges when attempting to differentiate treatment-related changes from recurrent tumor. In addition to basic MRI sequences, advanced MRI techniques, such as spectroscopic imaging and MR perfusion, may provide additional physiological information and should be considered in all equivocal situations. However, their interpretation can be more challenging due to a lack of standardization of image acquisition and processing (despite upcoming guidelines such as the European Society of Pediatric Oncology (SIOPE) Imaging Guideline), difficulties in interpretation (as a result of methodological problems), and a higher sensitivity to artifacts [31, 32].

In contrast, [^18^F]FET-PET, as an easy-to-read tool, might be particularly appealing to clinicians. It could substantively support clinical decisions, especially in cases where cMRI imaging is ambiguous.

Our retrospective study suffers from various limitations. It includes only 21 pediatric and adolescent patients with various tumor entities in different clinical settings. Thus, numbers are too small to differentiate among tumor types or clinical situations in which [^18^F]FET-PET might be of particular clinical value and large(r) multi-center studies are highly warranted. However, our cohort represents an authentic, real-world scenario encountered in daily routine.

PET-based tumor volumetry or a comparison of volumetry between PET and MR imaging was not performed. Additionally, no dynamic PET acquisitions which could have provided valuable diagnostic information [33–35] were performed. However, the reduction in acquisition time gained by static imaging must be acknowledged, as scans in this young patient group are often performed under general anesthesia or sedation in order to reduce motion artifacts. Single-session hybrid PET/MRI for the acquisition of both structural data and metabolic information should be considered for these patients, as the extra risk of additional anesthesia can be mitigated while obtaining optimized data co-registration.

Another limitation is the lack of stringent histopathological confirmation of imaging results, as tissue samples could not be obtained in all patients.

Nevertheless, the interpretation of [^18^F]FET-PET imaging is simple and robust, even in pediatric patients with CNS tumors for which pathophysiology and molecular pathogenesis are still not fully understood (and may differ from adult primary brain tumors). In this setting, a close collaboration with colleagues from pediatric oncology, surgery, neuroradiology, and neuropathology in multidisciplinary teams is of utmost importance.

Whereas cMRI remains the standard neuroimaging modality in pediatric patients with CNS tumors, the addition of amino acid PET may provide further information and subsequently influence treatment management, particularly in those select cases where standard neuro-oncological work-up is ambiguous.

## Conclusion

[^18^F]FET-PET is a robust imaging tool, which provides important additional information for treatment decisions in pediatric and adolescent patients with CNS tumors, especially in clinically challenging situations.

## Supplementary Information

Below is the link to the electronic supplementary material.

Supplementary file1 (DOCX 148 KB)

## Data Availability

All data and materials are available upon request.
