# Supplementary material for: [18F]FET-PET in children and adolescents with central nervous system tumors: does it support difficult clinical decision-making?
Source: Eur J Nucl Med Mol Imaging. 2023 Jan 21;50(6):1699–708. doi: 10.1007/s00259-023-06114-6 (PMC10119036; doi:10.1007/s00259-023-06114-6)
Supplement: Supplementary file 1 — Supplementary file1 (DOCX 148 KB) [file 259_2023_6114_MOESM1_ESM.docx]

**[^18^F]FET-PET/CT in children and adolescents with central nervous system tumors – Does it support difficult clinical decision making?**

Olivia Kertels, MD^1^, Jürgen Krauß, MD^2^, Camelia Maria Monoranu, MD^3^, Samuel Samnick, PhD^4^, Alexander Dierks, MD^4,5^, Malte Kircher, MD^4,5^, Milena I. Mihovilovic, MSc,^4^ Mirko Pham, MD^6^, Andreas K. Buck, MD^4^, Matthias Eyrich^7^, Paul-Gerhardt Schlegel^7^, Michael C. Frühwald, MD, PhD^8^, Brigitte Bison, MD^9*^, Constantin Lapa, MD^4,5*^

^1^ Institute of Diagnostic and Interventional Radiology, University Hospital Würzburg, Oberdürrbacher Strasse 6, 97080 Würzburg, Germany

^2^ Department of Neurosurgery, Section Pediatric Neurosurgery, University Hospital Würzburg, Oberdürrbacher Strasse 6, 97080 Würzburg, Germany

^3^ Department of Neuropathology, Institute for Pathology, University of Würzburg, Josef-Schneider-Strasse 2, 97080 Würzburg

^4^ Department of Nuclear Medicine, University Hospital Würzburg, Oberdürrbacher Strasse 6, 97080 Würzburg, Germany

^5^ Nuclear Medicine, Faculty of Medicine, University of Augsburg, Stenglinstrasse 2, 86156 Augsburg, Germany

^6^ Institute of Diagnostic and Interventional Neuroradiology, University Hospital Würzburg, Josef-Schneider-Str. 11, 97080 Würzburg, Germany

^7^ University Children’s Hospital, Department of Pediatric Hematology, Oncology and Stem Cell Transplantation, University of Würzburg, Josef-Schneider- Str. 2, 97080 Würzburg, Germany

^8^ Paediatric and Adolescent Medicine, University Medical Center Augsburg, Stenglinstrasse 2, 86156 Augsburg, Germany

^9^ Diagnostic and Interventional Neuroradiology, Neuroradiological Reference Center for pediatric brain tumor (HIT) studies of the German Society of Pediatric Oncology and Hematology, Faculty of Medicine, University of Augsburg, Stenglinstr. 2, 86156 Augsburg, Germany

^*^Both authors contributed equally.

**Corresponding author:**

Constantin Lapa, MD

Email: constantin.lapa@uk-augsburg.de

**Supplemental Material**

**Supplemental Table 1** Individual MRI results

|  | Age at ID/PET | Initial Diagnosis (ID) / Follow-Up (FU) | Medical History | Question to MRI | Answer | Reason | Technical Note |
| --- | --- | --- | --- | --- | --- | --- | --- |
| 1 | 3/3 | FU | EB, surgery and CTx, 2nd recurrence, surgical excision | Tumor residue or infarction along resection cavity | Tumor residue | Identical aspect as pre-operatively | Basic-MRI |
| 2 | 7/8 | FU | GBM, partial resection, RCTx | Regular follow-up | Confirmation of residual tumor, second manifestation => PD | Unchanged residual tumor new infratentoriell lesion | Basic-MRI |
| 3 | 3/11 | FU | DIPG long term survivor, pontine signal change 9 yrs after initial diagnosis | Vital tumor? | No sign of vital tumor | No change and no sign of aggressive tumor | Basic-MRI |
| 4 | 8/9 | ID | Headache 1-2 weeks before MRI, café-au-lait macules | Cause of symptoms? Tumor? | Tumor/ LGG | Diffusely infiltrating lesion of low cellularity | Basic-MRI |
| 5 | 13/14 | FU | AEP, biopsy at initial diagnosis, RCTx | Regular follow up, SD? PD? | Growing lesion, intense contrast enhancement PD? PsPD? Correlation with high dose region of irradiation needed | In contrast to GBM no tumor of high cellularity, inferior in discrimination between PsPD and PD | Basic-MRI |
| 6 | 15/15 | ID | MRI after traffic accident | Traumatic brain injury? | No traumatic lesion, incidental finding of aqueductal stenosis and LGG | Diffuse infiltration lesion of low cellularity | Basic-MRI |
| 7 | 7/7 | ID | Seizure (focal) | Cause of seizure? Tumor? | HGG | Tumor of high cellularity (restricted diffusion) and high proliferation (Cho:NAA ↑↑) | Basic-MRI + MRS |
| 8 | 2/4 | FU | EB, surgery RCTx as initial therapy, equivocal lesion along the resection cavity | Tumor residue, scar or new lesion? | Scar | Different aspect as primary tumor, no restricted diffusion | Basic-MRI |
| 9 | 14/14 | FU | 3 weeks of headache, vomiting and change of personality, partial resection | First FU after partial resection | Residual tumor, SD, GC/ HGG | Diffuse infiltrating tumor of high cellularity, involving 3 lobes | Basic-MRI |
| 10 | 13/23 | FU | Long history of slowly growing lesion, biopsy and RCTx | Regular follow up, SD? PD? | PD, initial diagnosis of GC | Diffuse infiltrating tumor, growing, now involving 3 lobes | Basic-MRI |
| 11 | 19/22 | FU | PMA of the optic pathway, partial resection, wait and see | Regular follow up, SD? PD? | PD | Tumor growth | Basic-MRI |
| 12 | 14/14 | FU | Partial tumor removal, no additional therapy | Regular follow up, SD? PD? | PD | Tumor growth, typical aspect of LGG | Basic-MRI |
| 13 | 8/17 | FU | Tectum glioma, biopsy, third ventriculostomy, PD during long follow up | Regular follow up, SD? PD? | PD | Tumor growth, typical aspect of LGG | Basic-MRI |
| 14 | 8/15 | FU | Intensified therapy for disseminated MB, pontine signal change on regular FU MRI | Second malignancy? Posttherapeutic change? | No sign of malignancy, more likely posttherapeutic changes | Minimal changes, no signs of high cellularity, proliferation, or perfusion (DIPG on FU) | Basic-MRI + MRS |
| 15 | 11/11 | FU | GBM 4 months after initial diagnosis, surgery, RCTx | Regular follow up=> growth PsPD/ PD | Unclear, shortly after irradiation | Growth and intensified contrast enhancement, MR-perfusion+MRS needed | Basic-MRI |
| 16 | 2/14 | FU | PNET, initial diagnosis and therapy in Moscow 12/06, new seizures | Regular follow up, CR? PD? | CR, new lesion: cavernoma | Typical MRI aspect | Basic-MRI |
| 17 | 8/10 | ID | Bithalamic AA, additional right frontal lesion | Regular follow up, second lesion diagnosis LGG/ HGG? | PD, bithalamic lesion of high cellularity, frontal lesion LGG | Growth of diffuse tumor bithalamic tumor with high cellularity, second lesion of low cellularity | Basic-MRI |
| 18 | 1/2 | FU | PA of the optic pathway | Regular follow up, SD? PD? | PR | Tumor shrinkage, typical aspect of OPG/ LGG | Basic-MRI |
| 19 | 2/14 | FU | PA of the mesencephalon, multiple PDs followed by chemotherapy, SEED-implantation, gammaknife-therapy | Regular follow up, SD? PD? | Unclear, volume increase, no chance to differenciate between therapy induced change and tumor growth | Diffuse reaction, differentiation in LGG difficult (typical PsPD/ PD criteria like elevated CBV or cholin are missing (in contrast to GBM) | Basic-MRI + MRS |
| 20 | 17/17 | ID | Clinically symptomatic, first evaluation | Diagnosis? | GC/ most likely HGG | External basic MRI, diagnosis of GC sure, cellularity unsure | Basic-MRI without DWI; DWI, Perfusion + MRS needed |
| 21 | 6/20 | FU | Secreting GCT 11/02, chemotherapy and irradiation, symptomatic 2nd lesion 06/16 | Cause of symptoms? Recurrence? Diagnosis of second malignancy? | Second malignancy, aggressive tumor, no recurrence | Parenchymal lesion of high cellularity | Basic-MRI |

AA, anaplastic astrocytoma; AEP, anaplastic ependymoma; CR, complete response; DIPG, diffuse intrinstic pontine glioma; EB, ependymoblastoma; FU, follow up; GBM, glioblastoma multiforme; GC, gliomatosis cerebri; GCT, germ cell tumor; ID, initial diagnosis; LGG, low grade glioma; MB, medulloblastoma; MRS, MR-spectroscopy; PA, pilocytic astrocytoma; PD, progressive disease; PMA, pilomyxoid astrocytoma; PNET, primitive neuroectodermal tumor; SD, stable disease

**Supplemental Table 2** Individual PET results

|  | Tumor Entity | WHO grade | Initial diagnosis | PET | SUV_max_ | SUV_mean_ | Bkg | TBR_max_ | TBR_mean_ |
| --- | --- | --- | --- | --- | --- | --- | --- | --- | --- |
| 1 | Ependymoblastoma | IV | No | Pos | 2.22 | 1.87 | 0.82 | 2.71 | 2.28 |
| 2 | GBM | IV | No | Pos | 3.33 | 3.00 | 1.20 | 2.78 | 2.50 |
| 3 | DIPG | IV | No | Neg | 1.7 | 1.51 | 1.12 | 1.52 | 1.35 |
| 4 | LGG | n/a | Yes | Neg | 1.63 | 1.35 | 1.09 | 1.50 | 1.24 |
| 5 | Anaplastic ependymoma | III | No | Pos | 5.8 | 5.19 | 1.55 | 3.74 | 3.35 |
| 6 | LGG | n/a | Yes | Neg | 1.27 | 1.09 | 1.29 | 0.98 | 0.84 |
| 7 | GBM | IV | Yes | Pos | 4.52 | 3.35 | 0.88 | 5.14 | 3.81 |
| 8 | Ependymoblastoma | IV | No | Neg | 1.41 | 1.28 | 1.01 | 1.40 | 1.27 |
| 9 | Anaplastic astrocytoma (GC) | III | No | Pos | 3.49 | 3.09 | 1.32 | 2.64 | 2.34 |
| 10 | Anaplastic astrocytoma (GC) | III | No | Pos | 6.14 | 5.39 | 1.37 | 4.48 | 3.93 |
| 11 | PMA (OPG) | II | No | Pos | 3.76 | 3.37 | 1.42 | 2.65 | 2.37 |
| 12 | GBM | IV | No | Neg | 2.39 | 2.01 | 1.45 | 1.65 | 1.39 |
| 13 | Pilocytic astrocytoma/ Tectumglioma | I | No | Pos | 2.54 | 2.28 | 1.11 | 2.29 | 2.05 |
| 14 | DIPG (second malignancy, initially medulloblastoma) | IV | No | Neg | 2.25 | 1.97 | 1.88 | 1.20 | 1.05 |
| 15 | GBM | IV | No | Pos | 5.74 | 4.24 | 1.23 | 4.67 | 3.45 |
| 16 | PNET | IV | No | Neg | 2.41 | 2.04 | 1.57 | 1.54 | 1.30 |
| 17 | Anaplastic astrocytoma | III | Yes | Pos | 3.88 | 3.12 | 1.50 | 2.59 | 2.08 |
| 18 | Pilocytic astrocytoma/ OPG (initially anaplastic astrocytoma) | I | No | Pos | 4.90 | 3.22 | 1.51 | 3.25 | 2.13 |
| 19 | Pilocytic astrocytoma | I | No | Pos | 2.16 | 1.83 | 0.85 | 2.54 | 2.15 |
| 20 | GBM (GC) | IV | Yes | Pos | 5.02 | 3.55 | 1.08 | 4.65 | 3.29 |
| 21 | Anaplastic astrocytoma (second malignancy, initially GCT) | III | No | Pos | 4.96 | 3.65 | 1.42 | 3.49 | 2.57 |

Bkg, SUV_mean_ of background; DIPG, diffuse intrinsic pontine glioma; ETMR, embryonal tumor with multilayered rosettes; GCT, germ cell tumor; GBM, glioblastoma; GC, gliomatosis cerebri; LGG, low grade glioma; n/a, not available; Neg, negative; OPG, optic pathway glioma; PD, progressive disease; PMA, pilomyxoid astrocytoma; PNET, primitive neuroectodermal tumor; Pos, positive; SUV, standardized uptake value; TBR, tumor-to-background ratio

**Supplemental Figure 1**


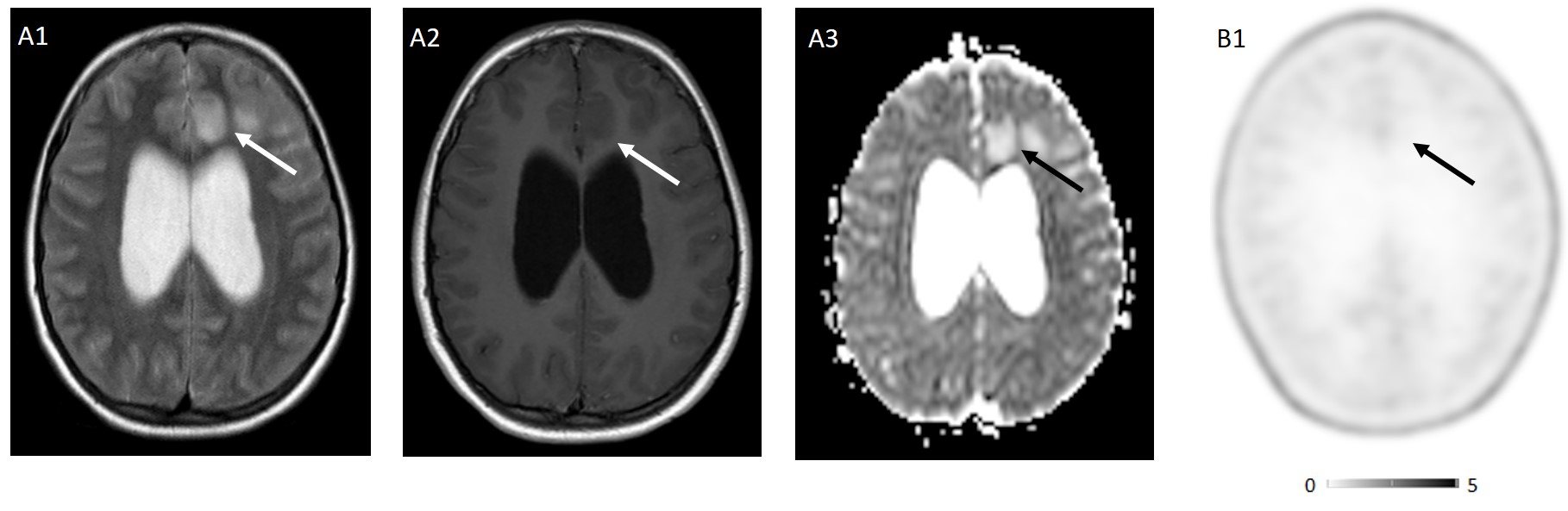


Example of a patient (Patient #6) with a newly diagnosed tumor in the left frontal lobe. Initial MRI shows high signal on T2WI (A1; white arrow), no contrast enhancement (A2; white arrow), and no restricted diffusion (DWI- Acquired Diffusion Coefficient, A3; black arrow). [^18^F]FET-PET/CT depicts no amino acid uptake in the left frontal tumoral region (B1; black arrow), consistent with low-grade glioma. As a result, no biopsy or surgery was performed. More than six years after initial diagnosis, the patient is still without therapy and alive to date
